# Supplementary material for: Polyhydroxybutyrate Rice Hull and Torrefied Rice Hull Biocomposites
Source: Polymers (Basel). 2022 Sep 17;14(18):3882. doi: 10.3390/polym14183882 (PMC9501343; doi:10.3390/polym14183882)
Supplement: Supplementary file 1 [file polymers-14-03882-s001.zip › polymers-1894369-supplementary.pdf]

polymers

MDPI

Supplementary Information

Table S1. Analysis of Variance Table.

	Df	Sum Sq	Mean Sq	F value	Pr(>F)
Tensile modulus	4	1,511,404	377,851	52.734	2.74E-08
Residuals	14	100,312	7,165		
Tensile strength	4	630.94	157.736	55.083	2.96E-08
Residuals	14	40.09	2.864		
Tensile elongation	4	15.49	3.8713	13.827	9.04E-05
Residuals	14	3.92	0.28		
Flexural modulus	4	90,751,735	22,687,934	98.435	1.39E-09
Residuals	13	2,996,335	230,487		
Flexural stress at yield	4	4,158.60	1,039.65	117.74	4.51E-10
Residuals	13	114.80	8.83		
HDT	4	651.20	162.801	11.97	8.97E-03
Residuals	5	68.00	13.601		

Df = degrees of freedom. **Sq** = square.
